# Dental erosion in French adolescents 

**DOI:** 10.1186/s12903-015-0133-4

**Published:** 2015-11-19

**Authors:** Michèle Muller-Bolla, Frédéric Courson, Violaine Smail-Faugeron, Thibault Bernardin, Laurence Lupi-Pégurier

**Affiliations:** Dental Faculty, University Nice Sophia Antipolis. CHUN, Nice, France; URB2i - EA 4462, University Paris Descartes, Montrouge, France; Dental Faculty, University Paris Descartes, Montrouge, France

## Abstract

**Background:**

Since the 2000s, different epidemiological studies focusing on the prevalence or the aetiology of DE in adolescents recognised them as an at-risk population due to their eating behaviours. None was carried out in French adolescents.

The primary objective of this study was to assess the prevalence of dental erosion (DE) using the total BEWE score among adolescents in the department of Alpes Maritimes, France. The secondary objectives were to observe changes in prevalence estimates depending on both the cutoffvalue of total BEWE score with different teeth/dental surfaces examined, and to identify the related risk factors.

**Methods:**

A cross-sectional study in a multistage random sample of 339 14-yr-old schoolchildren was carried out in 2014. The children completed a self-administered questionnaire concerning diet and oral habits. Caries was assessed with ICDAS-II (International Caries Detection and Assessment System-II) criteria and erosion with BEWE (Basic Erosive Wear Examination) index. The total BEWE score was calculated to assess the DE prevalence with two cutoff values (3 and 1). Data were analysed using descriptive statistics and logistic regression models.

**Results:**

The 331 children were aged 14.4 ± 0.5 years. The DE prevalence was 39 % using a total BEWE score ≥ 3. With a cutoff total BEWE score of 1 (at least one affected tooth), the prevalence varied from 3.9 to 56.8 % depending on the teeth/surfaces that were used for the analysis. The DE prevalence, assessed with only first molars and maxillary incisors, was about 54 %. The risk factors for DE (total BEWE score ≥ 3) were daily consumption of acidic beverages (OR: 4.0; 95 % CI: 2.1–7.6) and acidic sweets (OR: 3.2; 95 % CI: 1.2–8.0), low socio economic category (OR: 2.4; 95 % CI: 1.1–5.0) and visible dental biofilm (OR: 2.0; 95 % CI: 1.2–3.4).

**Conclusion:**

Depending on the method chosen, the prevalence varied from 3.9 to 56.8 % among these adolescents. Thus, a consensus on choice of index, teeth to examine and age at assessment is necessary to standardise measurement of DE prevalence.

## Background

Dental erosion (DE) is a non-carious lesion consisting of progressive and irreversible loss of dental hard tissue due to the chemical process of acid dissolution that does not involve bacterial plaque acid [1, 2]. Its overall clinical appearance may also involve a frictional component, such as abrasion or attrition, particularly in older age groups. Since the 2000s, different epidemiological studies focusing on the prevalence or the aetiology of DE in adolescents recognised them as an at-risk population due to their eating behaviours. The studies were carried out in the Americas [3–14], Arabian countries [15–23], an Asian country [24, 25] and in Western Europe [2, 26–35]. The prevalence of DE ranged from 7 to 95 % and, except in two studies, the extension of severe erosion into dentine was usually infrequent [11, 16] (Table 1). The wide variation of prevalence in teenagers is suggestive of the difficulty in finding a unanimously accepted index among researchers for measuring and detecting erosive lesions. A recent systematic review estimated an overall worldwide prevalence of tooth erosion of 30 % (95 % CI: 24–37) by mixing different clinical indices and without specifying the teeth examined. Futhermore, the review included population-based studies in the permanent teeth of children and adolescents aged 8–19 years [36].

**Table 1:** Prevalence of dental erosion in adolescents (12–16 years-old) assessed on a population-based samples (prevalence studies published since 2000)

Authors	Year	Country (Area)	Age (years)	Sample size	Erosion/Wear	Index used	Permanent teeth (surfaces) Examined	Prevalence (%)
El Karim et al. [23]	2007	Sudan (Khartoum)	12–14	157	Erosion	Smith and Knight Tooth Wear Index (TWI) [51].	UI (B, P)	66.9 %
Hamasha et al. [15]	2014	Jordan (Amman, Iibid, Al-Karak)	12–14	3 812	Erosion	TWI [51] modified by Millward et al. [52]: 0 (B/L/O/I) No loss of enamel surface characteristics. 1 (B/L/O/I) Loss of enamel surface characteristics. 2 (B/L/O) Loss of enamel, visible dentine for less than 1/3 of the surface. 2 (I) Loss of enamel just exposing dentine. 3 (B/L/O) Loss of enamel, visible dentine for > 1/3 of the surface. 3 (I) Loss of enamel and substantial loss of dentine. 4 (B/L/O) Complete loss of enamel, or pulp exposure, or exposure of secondary dentine. 4 (I) Pulp exposure or exposure of secondary dentine.	All permanent teeth (all surfaces)	32.2 %
Al Majed et al. [16]	2002	Saudi Arabia (Riyadh)	12–14	862	Erosion	Modified TWI derived from the 1998 United Kingdom Adult Dental Health Survey [53]: 0 No obvious wear facets in enamel. 1 Marked wear facets in enamel. Enamel only, Loss of surface characterisation. 2 Wear into dentin, dentin exposed occlusally/incisally. Loss of enamel exposing dentine. 3 Extensive wear into dentin, greater than 2 mm^2^ of exposed dentin, pulp exposure. Loss of enamel and dentin resulting in pulpal exposure.	UI (B, P), M1 (O)	95 % (DE in at least one tooth) 26 % (DE into dentine or into pulp)
McGuire et al. [3] Okunseri et al. [13]	2009 2011	United States (NHANES)	13–19	1 962	Erosion		Incisors, canines, M1 (O)	45.9 % (DE in at least one tooth): 13–14 year-old: 39.6 % 16–17 year-old: 44.5 %
Bardsley et al. [26] Milosevic et al. [27]	2004	Nord west England (Preston)	14	2 385	Wear	Simplified version of TWI dichotomised as the presence or absence of dentinal exposure (S-TWI) (1984): 0 No wear into dentin. 1 Dentine just visible (including cupping) or dentine exposed for less than 1/3 of surface. 2 Dentin exposure greater than 1/3 of surface. 3 Complete loss of enamel on a surface, exposure of pulp or secondary dentin.	Incisors, canines, M1 (O)	53 %
Bardolia et al. [31]	2010	UK (Isle of Man)	13–14	629	Erosion		Incisors, canines, M1 (O)	51 % (20 % if exclusion of incisal edge wear)
Abu-Ghazaleh et al. [22]	2013	Jordan (Amman)	15–16	1 602	Wear		Incisors, canines, M1 (O)	51 %
Dugmore et al. [32] Dugmore et al. [28]	2003 2004	UK (Leicestershire, Rutland)	12 14	1 753 1 308 re-examined	Erosion	Index of O’Brien [32]. Depth: 0 Normal enamel. 1 Loss of enamel surface characteristics. 2 Loss of enamel exposing dentine 3 Loss of enamel and dentine with pulp exposure. Area: 1 Less than one third of surface involved. 2 Between one and two thirds of surface involved. 3 More than two thirds of surface involved.	Incisors, M1	12 year-old: 59.7 % (2.7 % exhibiting exposed dentine) 14 year-old: 64.1 % (56.3 % at 12 year-old)
Sanhouri et al. [18]	2010	Sudan (Khartoum)	12–14	1 138	Wear		All permanent teeth (all surfaces)	74 %
Gurgel et al. [9, 10]	2011	Brazil (Bauru)	12, 16	414	Erosion		UI (B, P), M1 (O)	20 % (withoutloss of dentine)
Habib et al. [12]	2013	United States (Kansas city)	12	218	Erosion		UI (B, P)	10 %
Van Rijkom et al. [30]	2002	The Netherlands (The Hague)	10–13 15–16	345 400	Erosion	Modified scale of Lussi by Van Rijkom [54]: 0 No visible smooth wear. 1 Slight smooth enamel wear, surface with silky-shining, ‘melted’ appearance. 2 Deep smooth enamel wear, dentine is shining through (light yellow). 3 Smooth wear into dentine on L/P or O surfaces, or less than one-half of B surfaces (yellow). 4 Smooth wear into dentine on more than one-half of B surfaces.	All permanent teeth (all surfaces)	3 % of 10–13 year-olds 30 % of 15–16 year-olds
Truin et al. [33]	2005	The Netherlands (The Hague)	12	324	Erosion		UI, UC (P); M1 (O)	24 %
El Aïdi et al. [34]	2008	The Netherlands (Oss)	10–12	622	Erosion		All permanent teeth (all surfaces)	32.2 % (deep enamel erosion: 1.8 %)
Arnadottir et al. [29]	2003	Iceland (Reykjavik)	15	278	Erosion	Modified scale of Lussi (1991): 0 No erosion. 1 Loss of surface enamel, dentine not involved (anterior teeth), enamel erosion on cusp tips that cannot be attributed to attrition (posterior teeth). 2 Erosion extending into dentine. 3 Extending close to pulp (pulp chamber visible).	All permanent teeth (all surfaces)	21.6 %
Arnadottir et al. [2]	2010	Iceland	12, 15	1 507	Erosion		All permanent teeth (all surfaces)	15.7 % of 12-year-olds (*n* = 757) 30.7 % of 15-year-olds (*n* = 750)
Peres et al. [7]	2005	Brazil (Joaçaba)	12	391	Erosion	Dental erosion index of O’Sullivan [55]. Grade of severity: 0 Normal enamel. 1 Matte appearance of the enamel surface with no loss of contour. 2 Loss of enamel only. 3 Loss of enamel with exposure of dentin (ADJ visible). 4 Loss of enamel and dentin beyond ADJ. 5 Loss on enamel and dentin with exposure of pulp. Area of surface affected by erosion: Code- Less than half of the surface affected. Code + More than half of the surface affected.	UI	13 %
Correr et al. [4]	2009	Brazil (Piracicaba, São Paulo)	12	389	Erosion		UI, M1	26 %
Wang et al. [24]	2010	China (Guangzhou)	12–13	1 499	Erosion		Incisors, M1	27.3 %
Vargas-Ferreira et al. [11]	2011	Brazil (Santa Maria)	11–14	870	Erosion		UI, M1	7.2 %
Kumar et al. [17]	2013	Karnataka, South India	11–14	605	Erosion		All permanent teeth (all surfaces)	8.9 %
Aguiar et al. [8]	2014	Brazil (Campina Grande)	15–19	675	Erosion		UI, M1	21 %
Auad et al. [5] Waterhouse et al. [6]	2007 2008	South east Brazil (TrêsCorações)	13–14	458	Erosion	Criteria used in UK National Diet and nutrition survey [56]: 0 Normal. 1 Enamel only. Less than one third of surface involved. 2 Enamel and dentine. One third up to two thirds of surface involved. 3 Enamel, dentine and pulp. Two thirds or more of surface involved.	UI (B/P), M1 (O)	34.1 % (no evidence of erosion affecting dentine)
Huew et al. [19–21]	2011	Libya (Benghazi)	12	791	Erosion		UI (B/P), M1 (O)	40.8 %
Margaratis et al. [35]	2011	Greece (Attica)	14–16	502	Erosion	BEWE scoring system [46]: 0 No erosive tooth wear. 1 Initial loss of surface texture. 2 Distinct defect; hard tissue loss < 50 % of the surface area. 3 Hard tissue loss ≥ 50 % of the surface area.	All permanent teeth (all surfaces)	58 % (BEWE >0) If S-TWI on incisors, M1(O): 51.6 %
Zhang et al. [25]	2014	China (Hong Kong)	12	600	Erosion		All permanent teeth (all surfaces)	75 % (BEWE > 0) but no severe erosion (BEWE = 3)
Alvarez Loureiro L. et al. [14]	2015	Montevideo (Uruguay)	12	1 136	Erosion		All permanent teeth (B, P, O)	52.9 % (BEWE >0)

*B* Buccal, *I* Incisal, *P* Palatal, *L* Lingual, *O* Occlusal, *UI* upper incisors, *UC* upper canines, *M1* first molars, *UK* United Kingdom, *BEWE* Basic Erosive Wear Examination

When focusing on Europeans adolescents, the prevalence of DE has ranged from 18 to 64 % [28, 33] but no epidemiological study of DE has so far been carried out in France (Table 1). The prevalence of DE was only indirectly considered in the study of Bartlett et al. [37]; it focused on the prevalence of tooth wear in 18–35 year-old Europeans including France and six other countries. The index used to measure both severity and distribution of tooth wear was the Basic Erosive Wear Examination (BEWE), as recommended by different authors [1, 38–41] but the protocol did not consider all surfaces, just the buccal and lingual surfaces of all teeth [37]. Nevertheless early signs of erosion are often observed on the occlusal surfaces of molars [1]. The problem of which clinical index to use and which teeth and tooth surfaces to observe arises since many different methods are available in the literature (Table 1). Finally, no studies that address the risk indicators for DE in France have been found.

The primary objective of the present study was to assess the prevalence of DE using the total BEWE score in a sample of French adolescents. The secondary objectives were to observe changes in prevalence depending on both the cutoff value of total BEWE score on different teeth/dental surfaces examined, and to identify the related risk factors.

## Methods

This cross-sectional survey was carried out in 2014 in the Alpes Maritimes (AM) department in the south-east of France. Ethical approval was obtained from the local ethics committee (“Comité Protection Personnes sud Méditerranée V”).

### Study population

Alpes Maritimes is the 14th most densely populated department in France with 251 persons per sq km. It includes Nice, the fifth largest city in France. Four of the 71 public secondary schools are located in rural areas, the others are in urban areas where students enrolled in priority education schools (PES) (12.5 %) are of lower socioeconomic status than others.

### Sampling procedure

A minimum sample size of 322 adolescents was required assuming prevalence of DE of 30 % [42, 43], precision and level of statistical signification of 0.05. We used a two-stage, stratified sampling method to obtain a representative sample of approximately 14-yr-old adolescents from 8th grade high-schools by randomisation of the schools on the basis of geographic location (urban/rural) and socio-economic category (PES/not PES). A list of all the schools was obtained from the local education department. Firstly, a consent form to participate in the study was given to all of the parents prior to the dentist’s visit to the randomised schools. Secondly, the children who were present on the day of the examination and for whom positive parental and personal consent was obtained were included. Children were excluded if they were not 14 years old during the school year or if they were currently wearing orthodontic appliances. Children affected by a hereditary or acquired dental structural abnormality were also excluded.

### Data collection

The data were collected on the day of the examination using a self-completed questionnaire. This questionnaire was based on one used in previous studies that identified risk factors for DE [44] and included sociodemographic characteristics, dietary and oral health behaviours. The French version was first tested in 30 French 14-yr-olds and it was found to be comprehensible for this age group.

### Clinical examinations

Before examination, with good lighting (Power-Spotlight, Bisico, France), the teeth were carefully cleaned (Happy morning brushes, Hager & Werken, France) and dried (Trans’Care Max, Satelec group, Acteon equipment, France). Lesions were scored using the BEWE index (0 = no erosion, 1 = early enamel surface loss, 2 = enamel and dentine surface loss < 50 %, 3 = surface loss 50 %) on all permanent tooth scoreable surfaces. With this scoring system, all teeth were examined, and the most severely affected surfaces (buccal/labial, occlusal, and lingual/palatal) in each sextant were recorded. Each subject obtained a total BEWE score that corresponded to the sum of the highest scores in the six sextants; and the prevalence of erosion was calculated on the basis of a total BEWE score ≥ 3. The risk level of DE is low when the total score is between 3 and 8; medium, between 9 and 13; and high for a score of 14 and above [38, 39, 41]. Carious lesions were recorded using the ICDAS-II (International Caries Detection and Assessment System) advanced method on all scoreable surfaces of permanent teeth. Children were affected by dentinal lesions (ICDAS 4–6) or with both enamel and dentinal lesions (ICDAS 1–6) [45]. After the examination, the children received a card to take home reporting their dental needs.

All clinical examinations were carried out by a single examiner (TB) who was trained and calibrated by a paediatric dentist (MMB). A range of different levels of dental erosion was used in the calibration process, which was based on photographic images. Reliability was assessed through the Kappa test.

### Statistical analysis

Prevalence was estimated as the proportion that had a total BEWE score ≥ 3 considering highest scores in sextants (primary outcome). The proportions with a total BEWE score ≥ 1 on particular permanent teeth (incisors, canines, first molars) were secondary outcomes. Data analysis included comparisons of means and the Chi-squared test to investigate the univariate associations between the dichotomous dependent variable DE (primary outcome) and a range of demographic, dietary and oral care variables. Independent variables which were associated with DE (*p* < 0.1) were entered as candidate variables into a stepwise multiple logistic regression analysis. All the significant variables were ranked using a multivariable logistic regression analysis. Similar multivariable logistic regression analyses were tested, changing the cutoff value of the dependent variable (total BEWE score ≥ 1 considering all surfaces). The level of statistical significance was set at 0.05. In each classroom, one child chosen at random was examined twice one week later by the same examiner using the ICDAS-II advanced method and the BEWE index to measure intra-examiner reliability. The corresponding Kappa values assessed in 34 re-examined children one week later were respectively 0.8 (ICDAS-II) and 0.9 (BEWE). Data were analysed using SPSS19.0.

## Results

Of the 495 subjects invited to participate, 143 did not return the parental consent, 3 were absent on the day of the visit dates and 18 were excluded. The 331 children were aged 14.4 ± 0.5 years. There were 174 girls and 157 boys included; 12.1 % (*n* = 40) were enrolled in PES and 5.1 % (*n* = 17) in rural schools.

The highest total BEWE score ≥ 3 (cumulative score of all sextants) in our study was in the range 9–13 (medium risk level) for only one adolescent. When the total BEWE cutoff score was ≥ 3, prevalence was 39 % (primary objective). When the cut off value was 1, i.e. at least one tooth with signs of DE, the prevalence depended on the dental surfaces observed. If only maxillary incisors were examined, as in the method used in some studies carried out in adolescents [7, 12, 23] (Table 1), the prevalence decreased from 39 to 3.9 %. Conversely, if first molars were examined in addition to the maxillary incisors, whatever the surfaces considered, the prevalence was about 54 %, close to 56.8 % for all the surfaces examined on all permanent teeth (Table 2). These results depend on the distribution of the DE lesions. Light extension (BEWE 3–8) was more frequent in mandibular (16.9) compared with maxillary (9.0) teeth. The BEWE score 3 was found just once on the occlusal subsurface of the second maxillary right premolar and score 2 was found on occlusal surfaces of permanent molars and second premolars. First molars, and more particularly the lower ones, were the most affected teeth (Table 3). If we consider the results for each kind of tooth, 98.0 % of the subjects with at least one second molar affected by DE presented also DE on the first molars. Second premolars were more often affected than both the first ones and the second molars.

**Table 2:** Dental Erosion prevalence in a sample of French adolescents (*n* = 331) according to the dental surfaces of permanent teeth examined

Dental surfaces examined	BEWE	Prevalence
Maxillary incisors ^a^	Total BEWE ≥ 1	3.9 %
Maxillary incisors and occlusal surfaces of first molars ^b^	Total BEWE ≥ 1	54.4 %
Maxillary incisors and first molars on all scorable surfaces ^c^	Total BEWE ≥ 1	54.4 %
Incisors and first molars ^d^	Total BEWE ≥ 1	55.0 %
Six upper and lower anterior teeth, occlusal surfaces of first molars ^e^	Total BEWE ≥ 1	55.0 %
All teeth (all surfaces) ^f^	Total BEWE ≥ 1	56.8 %

^a^ [7, 12, 23], ^b^ [5, 6, 9, 10, 16, 19–21], ^c^ [4, 8, 11], ^d^ [24, 28, 32], ^e^ [3, 13, 22, 26, 31], ^f^ [2, 14, 15, 17, 18, 25, 29, 30, 34, 35]. *BEWE* Basic Erosive Wear Examination

**Table 3:** BEWE scores according to affected teeth in 331 subjects

		17–27	16–26	15–25	14–24	13–23	12–22	11–21	31–41	32–42	33–43	34–44	35–45	36–46	37–47
Score 1	B	1 (0.2)	22 (3.3)	10 (1.5)	8 (1.2)	21 (3.2)	12 (1.8)	17 (2.6)	8 (1.2)	5 (0.8)	5 (0.8)	8 (1.2)	10 (1.5)	16 (2.4)	3 (0.5)
	O	4 (0.6)	180 (22.7)	81 (12.2)	13 (2.0)							32 (4.8)	133 (20.1)	237 (35.8)	43 (6.5)
	L		3 (0.5)		1 (0.2)	4 (0.6)	2 (0.3)	2 (0.3)			2 (0.3)			5 (0.8)	
Score 2	B													1 (0.2)	
	O	4 (0.6)	30 (4.5)	8 (1.2)								1 (0.2)	17 (2.6)	93 (14.1)	10 (1.5)
	L														

*B* Buccal, *O* occlusal, *L* lingual, *BEWE* Basic Erosive Wear Examination

The risk factors for DE (Total BEWE score ≥ 3) were, in decreasing order, the consumption of acidic beverages and sweets, and the low socio-economic category of participants. Area of residence, the presence of carious lesions (ICDAS 1–6 or 4–6) and prolonged retention of drinks in the mouth were not significantly associated with erosive experience in adjusted logistic regression analyses; visible dental biofilm was only associated if lesions ICDAS 4–6 were included in the multivariable logistic regression analysis (Table 4). Thus the consumption of acidic beverages (OR: 4.0; 95 % CI: 2.1–7.6) and sweets (OR: 3.2; 95 % CI: 1.2–8.0), low socio-economic category (OR: 2.4; 95 % CI: 1.1–5.0) and the presence of visible dental biofilm (OR: 2.0; 95 % CI: 1.2–3.4) had an effect on the primary outcome. When changing the dependent variable by substitution of primary outcome with the total BEWE score ≥ 1 considering all surfaces, independent variables which were entered as candidate variables into a stepwise multiple logistic regression analysis were different (Table 5). With this cutoff value, only acidic beverages (OR: 6.4; 95 % CI: 2.9–14.0) and drinking method (OR: 3.5; 95 % CI: 1.4–8.9) had an effect on the dependant variable.

**Table 4:** Association between erosive experience (total BEWE ≥ 3) and independent variables. Unadjusted and adjusted logistic regression analyses

Independent variables		number	Erosive experience (Total BEWE ≥ 3)				
			No *n* (%)	Yes *n* (%)	Unadjusted OR (95 %)	Adjusted OR (95 %)	Adjusted OR (95 %)
Socio-economic category	No PES	291	186 (64)	105 (36)	1	1	1
	PES	40	16 (40)	24 (60)	2.7 (1.4–5.2)	2.2 (1.1–4.7)	2.2 (1.1–4.7)
Area of residence	Rural	17	6 (35)	11 (65)	1	1	1
	Urban	314	196 (62)	118 (38)	0.3 (0.1–0.9)	0.4 (0.1–1.1)	0.3 (0.1–1.0)
Sex^1^	Female	174	110 (63)	64 (37)	1	Not included	Not included
	Male	157	92 (59)	65 (41)	1.2 (0.8–1.9)		
Dental biofilm	No visible	247	163 (66)	84 (34)	1	1	1
	Visible	84	39 (46)	45 (55)	2.2 (1.4–3.7)	1.8 (1.1–3.3)	1.8 (1.0–3.1)
ICDAS 4–6 carious lesions	No	285	183 (64)	102 (36)	1	1	Not included
	Yes	46	19 (41)	27 (59)	2.6 (1.4–4.8)	1.3 (0.6–2.7)	
ICDAS 1–6 carious lesions	No	172	118 (69)	54 (31)	1	Not included	1
	Yes	159	84 (53)	75 (47)	2.0 (1.3–3.1)		1.4 (0.9–2.4)
Fluoride toothpaste^c^ ^1^	No	61	41 (67)	20 (33)	1	Not included	Not included
	Yes	270	161 (60)	109 (40)	1.4 (0.8–2.5)		
Dentist control during the past year ^1^	No	73	42 (57)	31 (43)	1	Not included	Not included
	Yes	258	160 (62)	98 (38)	0.8 (0.5–1.4)		
Acid reflux/repeated vomiting ^1^	No	295	181 (61)	114 (39)	1	Not included	Not included
	Yes	36	21 (58)	15 (42)	1.1 (0,6–2.3)		
Daily acidic beverage^a^	No	270	184 (68)	86 (32)	1	1	1
	Yes	61	18 (29)	43 (71)	5.1 (2.8–9.4)	3.8 (2.0–7.3)	3.6 (1.9–7.0)
Daily energy/sports drinks^b^ ^1^	No	326	200 (61)	126 (39)	1	Not included	Not included
	Yes	5	2 (40)	3 (60)	2.4 (0.4–14.5)		
Daily aicidic sweets	No	299	195 (65)	104 (35)	1	1	1
	Yes	32	7 (22)	25 (78)	6.7 (2.8–16.0)	2.9 (1.1–7.5)	3.0 (1.2–7.6)
Daily acid fresh fruits ^1^	No	277	172 (62)	105 (38)	1	Not included	Not included
	Yes	54	31 (57)	23 (43)	1.3 (0.7–2.4)		
Retained drink in the mouth after drinking	No	298	189 (63)	109 (37)	1	1	1
	Yes	33	13 (39)	20 (61)	2.7 (1.3–5.6)	1.8 (0.8–4.2)	1.9 (0.8–4.3)
Daily vitamin C	No	298	178 (60)	120 (40)	1	Not included	Not included
	Yes	33	24 (73)	9 (27)	0.6 (0.3–1.2)		

All consumers drank acidic beverages during and between meals: 94 % of them were sugared and the most consumed beverages were Cola (89 %)^a^ and Powerade (71 %)^b^. 1 (9.4 %) or 2 (90.3 %) daily tooth brushing^c^. ICDAS 4–6 or ICDAS 1–6 carious lesions were included in the adjusted logistic regression analyses. ^1^p > 0.1

**Table 5:** Association between erosive experience (total BEWE ≥ 1) and independent variables. Unadjusted and adjusted logistic regression analyses

Independent variables		number	Erosive experience (Total BEWE ≥ 3)				
			No *n* (%)	Yes *n* (%)	Unadjusted OR (95 %)	Adjusted OR (95 %)	Adjusted OR (95 %)
Socio-economic category	No PES	291	134 (46)	157 (54)	1	1	1
	PES	40	9 (23)	31 (77)	2.9 (1.4–6.4)	2.2 (1.0–5.2)	2.2 (0.9–5.1)
Area of residence	Rural	17	4 (24)	13 (76)	1	1	1
	Urban	314	139 (44)	175 (56)	0.4 (0.1–1.2)	0.4 (0.1–1.4)	0.4 (0.1–1.3)
Sex^1^	Female	174	78 (45)	96 (55)	1	Not included	Not included
	Male	157	65 (41)	92 (59)	1.2 (0.7–1.8)		
Dental biofilm	No visible	247	118 (48)	129 (52)	1	1	1
	Visible	84	25 (30)	59 (70)	2.2 (1.3–3.7)	1.8 (1.0–3.3)	1.7 (0.9–3.1)
ICDAS 4–6 carious lesions	No	285	132 (46)	153 (54)	1	1	Not included
	Yes	46	11 (24)	35 (76)	2.7 (1.3–5.6)	1.4 (0.6–3.1)	
ICDAS 1–6 carious lesions	No	172	90 (52)	82 (48)	1	Not included	1
	Yes	159	53 (33)	106 (67)	2.2 (1.4–3.4)		1.6 (1.0–2.47)
Fluoride toothpaste^1^	No	61	34 (56)	27 (44)	1	1	1
	Yes	270	109 (40)	161(60)	1.9 (1.1–3.3)	1.9 (1.0–3.6)	1.9 (1.0–3.7)
Dentist control during the past year ^1^	No	73	32 (44)	41 (56)	1	Not included	Not included
	Yes	258	111 (43)	147 (57)	1.0 (0.6–1.7)		
Acid reflux/repeated vomiting ^1^	No	295	130 (44)	165 (56)	1	Not included	Not included
	Yes	36	13 (36)	23 (64)	1.4 (0,7–2.9)		
Daily acidic beverage	No	270	135 (50)	135 (50)	1	1	1
	Yes	61	8 (13)	53 (87)	6.6 (3.0–14.5)	4.8 (2.1–10.9)	4.5 (2.0–10.2)
Daily energy/sports drinks^1^	No	326	142 (44)	184 (56)	1	Not included	Not included
	Yes	5	1 (20)	4 (80)	3.1 (0.3–27.9)		
Daily aicidic sweets	No	299	140 (47)	159 (53)	1	1	1
	Yes	32	3 (9)	29 (91)	8.5 (2.5–28.5)	2.9 (0.8–10.4)	2.9 (0.8–10.5)
Daily acid fresh fruits ^1^	No	277	124 (45)	153 (55)	1	Not included	Not included
	Yes	54	19 (35)	35 (65)	1.5 (0.8–2.7)		
Retained drink in the mouth after drinking	No	298	137 (46)	161 (54)	1	1	1
	Yes	33	6 (18)	27 (82)	3.8 (1.5–9.6)	2.9 (1.1–7.7)	2.9 (1.1–7.9)
Daily vitamin C	No	298	123 (41)	175 (59)	1	1	1
	Yes	33	20 (61)	13 (39)	0.5 (0.2–1.0)	0.5 (0.2–1.0)	0.5 (0.2–1.1)

## Discussion

The prevalence of DE using a total BEWE score ≥ 3 in a stratified sample of French adolescents was 39 %. This was within the confidence interval of the overall estimated prevalence of DE in European countries (33 %, 95 % IC 25–42) and outside the confidence interval of the overall worldwide prevalence of DE of 30 % (95 % IC: 24–37), both reported in a recent systematic review [36]. Except in the two studies carried out in the United States [3, 13, 14], prevalences in America ranged from 7.2 to 34.1 % and were lower than in France [4–12] whereas the systematic review, which included fewer studies, did not indicate any significant difference between American and European countries [36]. Conversely, except for the two studies with equivalent rates [15, 17, 19–21], four descriptive studies carried out in Arab countries indicated a higher prevalence, ranging from 51 to 95 % [16, 18, 22, 23]. Finally, by increasing order of prevalence in European countries, France was situated between The Netherlands [30, 33, 34] and Iceland [2, 29], with rates around 20–30 %, and United Kingdom [26–28, 31, 32] or Greece [35] around 50–60 %.

This ranking by country must be considered with caution as the prevalence of DE in adolescents was studied using different clinical indices. As indicated in the systematic review of Salas et al. [36], the prevalences of DE assessed with O’Sullivan’s or Lussi’s modified scales were lower [2, 4, 7, 8, 11, 17, 24, 29, 30, 33, 34] than in the present study using the total BEWE score. On the contrary, the prevalences of DE were higher with the Tooth Wear Index (TWI) modified three times (Table 1). One of them [31] easily explains these observations because this was dichotomised as the presence or absence of dentinal exposure [22, 26, 27, 31]. In the present study, the erosive lesions involved mostly the enamel. Only the study of Hamasha et al. [15], that used the TWI modified by Millward, did not confirm these result due to the lower prevalence. In France, the prevalence was assessed with BEWE total score because it is now both the most recommended index and the more recently used, especially in adults [35, 39, 41, 46, 47]. In children and adolescents, this was only used in a retrospective study [40] and in a study with a population-based sample mixing adults and adolescents [48]. However, three prevalence studies focusing on adolescents [14, 25, 35] have used BEWE scores but with a cutoff value of 1 for DE diagnosis (Table 1). They reported prevalences higher than 50 %, as in our study, and an increase of the prevalence from 39 to 56.8 % by changing the cutoff value (Table 2). There is therefore a problem of both index choice and cut off value, not forgetting the type of tooth wear registered. Studies considering erosion, attrition (wear resulting from tooth to tooth grinding) and abrasion (wear resulting from tooth to other hard surfaces) showed higher prevalences [18, 22, 26, 27] compared with others (Table 1). This could be explained by an easier differential diagnosis in the young population than in the adult one [2, 24, 49]. Finally, the kind of tooth examined influenced the DE prevalence: the examination of maxillary incisors decreased significantly the DE prevalence whereas there was no significant difference between other situations (Table 2). Our results confirm those of other studies, since erosion was found to be greater in posterior than in anterior teeth and the most frequently affected teeth were the lower first molars [2, 3, 16, 17, 22, 31, 33] (Table 3). The most common clinical manifestation of DE was the appearance of cup-like lesions on the cusp tip of lower first molars. By contrast, other studies have recorded DE mainly on anterior teeth [4, 5, 9–11, 24, 28, 29] where loss of smooth surface enamel is more difficult to see [50]. Yet our examination conditions in schools were optimal due to adequate light and drying facilities. The absence of DE on mandibular incisors in Table 2 is due to their protection from acid attack by the high flow of submandibular saliva [5]. Thus the examination of upper incisors alone and first molars appears sufficient in prevalence studies carried out in adolescents at an age to be fixed by consensus. In the present study, the age of 14 years was used for examination of all teeth due to exposition to possible intrinsinc and extrinsinc aetiological factors for some time; if examining upper incisors and first molars can be considered sufficient, adolescents could be examined at the age of 12. This age should allow both DE prevalence and dental caries to be registered in the same study. Regarding severity, the first degree involving the enamel alone was most common, as has been reported in most of the studies in adolescents [4–7, 10–12, 19–21, 28, 30, 32, 34, 49]. To conclude this first part focusing on prevalence, the limitations of the present study should be considered. The study sample was not nationally representative. As the majority of prevalence studies [4–8, 10–12, 16–31, 33–35] (Table 1), it was a stratified sample in a particular area fairly representative of the target population. Thus the application of the results to the French population needs to be confirmed.

Dental erosion is considered a multifactorial condition. Because the present study was cross-sectional and not longitudinal, the design only permitted an analysis of the association between known risk factors and experience of DE in French adolescents to highlight eventual particular cultural behaviors. As in previous studies [17, 24], no significant difference in the prevalence of DE between urban and rural areas was found in adjusted logistic regression analyses. Contrary to the majority of the studies [3, 5, 8–11, 16–18, 25, 28, 33, 34], socio-economic status was associated with the experience of DE: the present study confirmed a significantly higher DE prevalence in the lowest social category [12, 24, 26, 32, 35, 49] more often cited than the contrary [7, 23]. The use of different indices could again explain these different results, because the significance of the association differed according the indices used in the same study [35]. Indeed socio-economic status had no effect on the dependant variable when a cutoff value of 1 was chosen (Table 5). Sex was not associated with DE prevalence, a result that is in accordance with numerous studies [4, 5, 7, 9–12, 17, 18, 25, 34, 35]. This may be explained by a similar pattern of exposure to risk factors in both sexes. A higher prevalence in males was found more particularly in studies which considered all tooth wear [22, 26, 30] and it was explained by differences in muscular strength and biting forces [26]. However, others studies targeting only DE found significant differences between the sexes [2, 3, 24, 28, 29, 31, 33] and one study showed a higher prevalence in girls [24].

Concerning oral health, the significantly greater occurrence of DE in the presence of carious lesions ICDAS 1–6 or 4–6 was inconsistent because the relationships were not statistically significant in adjusted logistic regression analyses (Table 4). This is in agreement with different studies using the DMFT (Decayed, Missing, Filled Teeth) index as independent variable [5, 11, 20, 31, 33]. However, caries experience was greater [25, 28] or lesser in adolescents with erosive experience [22] according to the studies. These different results could be explained by consumption habits of acidic beverages, sugar-free (light) or regular soda drinks. In the present study, this tendency to proportional relationship between carious lesions and DE could be explained by a higher proportion of participants who consumed acid and sugar-containing cola beverages. The inconsistent relationship between DE and cavitated carious lesions can be explained by the inclusion of visible dental biofilm in one adjusted logistic regression analysis (Table 4). In all cases, the associations were close to statistical significance (Tables 4 and 5). Usually, dental biofilm protects enamel from erosive lesions, especially on anterior teeth. In contrast, the main consumption of both sugary and acidic beverages or sweets in the present study increased the quantity of dental plaque, which was significantly associated with higher DE prevalence. As in other studies [3, 12, 15, 16, 22, 24, 30], no statistically significant association was observed between oral behaviours (daily toothbrushing with fluoride toothpaste, dental examination during the past year) and DE. Only the study of Bardolia et al. [31] showed that a brushing twice a day increased the risk of DE.

Thus, diet plays a major role. In agreement with the majority of studies in adolescents since the 2000s, strong associations were found between DE and acidic beverages [6, 12, 15, 17, 20, 23–25, 30, 31] or sweets [17, 18, 23] (Table 4). However, the acidic beverage was the sole risk factor to have an effect on the DE, whatever the cutoff value of total BEWE score (Tables 4 and 5). In France, this could be due to a parent education problem about oral health because drinks and food dispensers have been prohibited in schools since 2005. The method of drinking, keeping or not the beverage in the mouth, was also associated with DE [13, 15–17, 30]. Only two studies did not show these associations [4, 11]. In the present study, the method of drinking had an effect on the DE for a cutoff value of 1. The frequent behaviour of children of retaining a drink in the mouth could be explained by a particular position of the tongue, the higher DE rates on second premolars compared with first premolars. While there are conflicting results from different studies, the present work confirms the most frequent results for sports drinks [6, 8, 9, 19, 24, 29], fresh fruits [6, 8, 9, 13, 24, 31] and daily vitamin C [9, 17] which were not associated with DE. Only one study (sports drinks; [15]) and four studies (fresh fruits; [17–19, 22]) showed contrary results; in the case of fresh fruits, this concerned only oranges [22], lemon [17] or banana [19]. The present study confirmed that acid reflux and repeated vomiting were not associated with DE in adolescents [9, 11, 14, 24, 29]. Only two studies, both using TWI modified indices (which over assess DE), reported the contrary [15, 16]. This setting can sometimes be difficult to evaluate due to its subjectivity.

## Conclusion

The DE prevalence in Alpes Maritimes (France) was estimated to reach 39 %. It is difficult to compare this estimate with other national prevalences due to the wide range of indices, choice of teeth and age used in different studies. Although the BEWE index has been recommanded, the cut-off value of 1 still needs to be stipulated, for comparison with recent studies. The examination of maxillary incisors and first permanent molars appeared sufficient for assessing DE prevalence. Finally, the age of examination should be discussed because using 12-year-olds would have the advantage of allowing a prevalence study of both DE and caries prevalence.

## Abbreviations

DE: Dental erosion
BEWE: Basic Erosive Wear Examination
AM: Alpes Martimes
PES: Priority education schools
ICDAS: International Caries Detection and Assessment System
TWI: Tooth wear index
DMFT Decayed: missing, filled teeth
OR: Odds ratio
95 % CI: 95 % confidence interval
